# Mosaic Activating Mutations in *GNA11* and *GNAQ* Are Associated with Phakomatosis Pigmentovascularis and Extensive Dermal Melanocytosis

**DOI:** 10.1016/j.jid.2015.11.027

**Published:** 2016-04

**Authors:** Anna C. Thomas, Zhiqiang Zeng, Jean-Baptiste Rivière, Ryan O’Shaughnessy, Lara Al-Olabi, Judith St.-Onge, David J. Atherton, Hélène Aubert, Lorea Bagazgoitia, Sébastien Barbarot, Emmanuelle Bourrat, Christine Chiaverini, W. Kling Chong, Yannis Duffourd, Mary Glover, Leopold Groesser, Smail Hadj-Rabia, Henning Hamm, Rudolf Happle, Imran Mushtaq, Jean-Philippe Lacour, Regula Waelchli, Marion Wobser, Pierre Vabres, E. Elizabeth Patton, Veronica A. Kinsler

**Affiliations:** 1 Genetics and Genomic Medicine, UCL Institute of Child Health, London, UK; 2 MRC Institute of Genetics and Molecular Medicine, MRC Human Genetics Unit & Edinburgh Cancer Research UK Centre, Edinburgh, UK; 3 Equipe d’Accueil 4271, Génétique des Anomalies du Développement, University of Burgundy, Dijon, France; 4 Livingstone Skin Research Unit, UCL Institute of Child Health, London, UK; 5 Paediatric Dermatology, Great Ormond Street Hospital for Children, London, UK; 6 Department of Dermatology, Nantes University Hospital, Nantes, France; 7 Dermatology, Hospital Universitario Ramón y Cajal, Madrid, Spain; 8 Dermatology, Saint-Louis Hospital, Paris, France; 9 General Paediatrics, Robert-Debré Hospital, Paris, France; 10 Dermatology, University Hospital of Nice, Nice, France; 11 Neuroradiology, Great Ormond Street Hospital for Children, London, UK; 12 Dermatology, Regensburg University Clinic, Regensburg, Germany; 13 Paediatric Dermatology, Necker Enfants-Malades Hospital, Paris, France; 14 Dermatology, University Hospital Wuerzburg, Wuerzburg, Germany; 15 Dermatology, Freiburg University Medical Center, University of Freiburg, Freiburg, Germany; 16 Paediatric Urology, Great Ormond Street Hospital for Children, London, UK; 17 Dermatology, Dijon University Hospital, Dijon, France

**Keywords:** DNA, deoxyribonucleic acid, PPV, phakomatosis pigmentovascularis, SWS, Sturge-Weber syndrome, WT, wild type

## Abstract

Common birthmarks can be an indicator of underlying genetic disease but are often overlooked. Mongolian blue spots (dermal melanocytosis) are usually localized and transient, but they can be extensive, permanent, and associated with extracutaneous abnormalities. Co-occurrence with vascular birthmarks defines a subtype of phakomatosis pigmentovascularis, a group of syndromes associated with neurovascular, ophthalmological, overgrowth, and malignant complications. Here, we discover that extensive dermal melanocytosis and phakomatosis pigmentovascularis are associated with activating mutations in *GNA11* and *GNAQ*, genes that encode Gα subunits of heterotrimeric G proteins. The mutations were detected at very low levels in affected tissues but were undetectable in the blood, indicating that these conditions are postzygotic mosaic disorders. In vitro expression of mutant *GNA11*^*R183C*^ and *GNA11*^*Q209L*^ in human cell lines demonstrated activation of the downstream p38 MAPK signaling pathway and the p38, JNK, and ERK pathways, respectively. Transgenic mosaic zebrafish models expressing mutant *GNA11*^*R183C*^ under promoter *mitfa* developed extensive dermal melanocytosis recapitulating the human phenotype. Phakomatosis pigmentovascularis and extensive dermal melanocytosis are therefore diagnoses in the group of mosaic heterotrimeric G-protein disorders, joining McCune-Albright and Sturge-Weber syndromes. These findings will allow accurate clinical and molecular diagnosis of this subset of common birthmarks, thereby identifying infants at risk for serious complications, and provide novel therapeutic opportunities.

## Introduction

**Figure 1:**
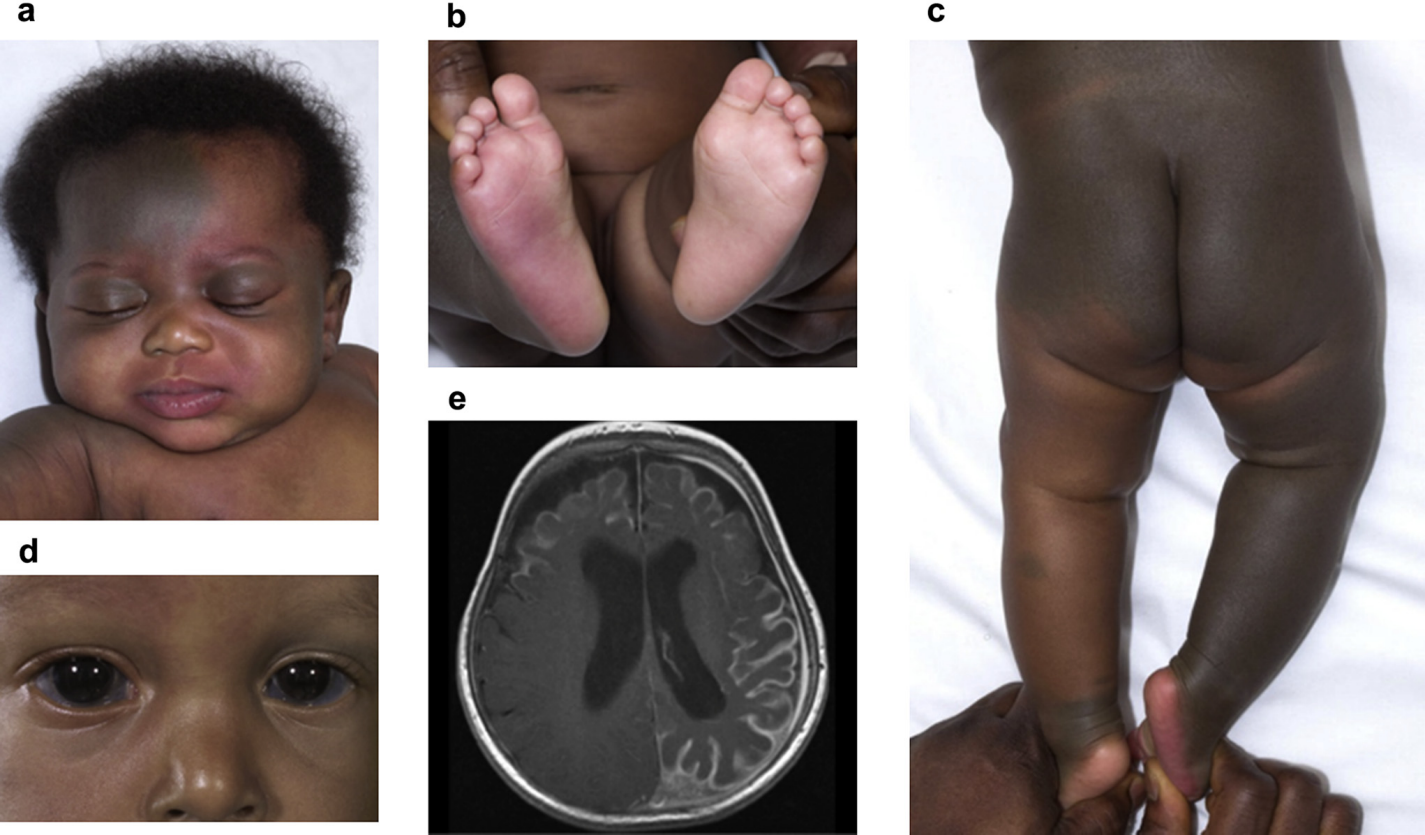
**Clinical examples of dermatological, ophthalmological, and neurological aspects of phakomatosis pigmentovascularis.** (**a–c**) Dermal melanocytosis and capillary malformation (port wine stain type) on the face, extensive dermal melanocytosis on the back and legs, and capillary malformation on the sole of the right foot. (**d**) Bilateral scleral melanocytosis with bilateral glaucoma. Capillary malformation and hemihypertrophy are just visible on right side of face. (**e**) Axial T1-weighed magnetic resonance image of the brain at the level of the lateral ventricles after administration of intravenous gadolinium contrast agent showing bilateral and asymmetrical thickening and enhancement of the pia mater. (Written consent for publication obtained in all cases.)

Mongolian blue spots (or, more appropriately, dermal melanocytosis) are common birthmarks, seen in up to 95% of African neonates and approximately 10% of white Caucasians (Cordova, 1981). As a result they are easily overlooked as a possible sign of underlying genetic disease. Classically they are flat, dark-blue lesions with indistinct edges overlying the buttocks and lower back, which undergo spontaneous resolution over a period of years. However, the appearances and behavior are not always classic (Gupta and Thappa, 2013). Some dermal melanocytosis involve unusual sites, cover large areas of the body surface, are more deeply pigmented, are better defined, and persist rather than resolve. These more atypical patterns of dermal melanocytosis can be associated with anomalies such as colocalized cleft lip (Igawa et al., 1994), ocular melanocytosis (Teekhasaenee et al., 1990), ocular melanoma (Shields et al., 2013), lysosomal storage disorders (Hanson et al., 2003), or vascular birthmarks. This latter association then falls under the diagnostic group of phakomatosis pigmentovascularis (PPV) (for clinical examples see Figure 1). Phakomatosis pigmentovascularis is a group of sporadic disorders of unknown frequency, defined by the co-occurrence of pigmentary and vascular birthmarks and subclassified clinically by the exact cutaneous phenotypes (Supplementary Table S1 online) (Happle, 2013, Hasegawa and Yasuhara, 1979; Ota et al., 1947). Whether the subtypes are distinct disorders or variable expressions of a single disorder has not been clear as the molecular and developmental pathogenesis of PPV has been unknown. The hypothesis of non-allelic twin spotting was previously expounded to explain the co-occurrence of two disparate birthmarks (Danarti and Happle, 2003) but was recently retracted by the author because of lack of supporting molecular evidence (Happle, 2013). Extracutaneous associations of PPV can be severe, including scleral or intraocular melanocytosis (Shields et al., 2011), glaucoma (Henry et al., 2013, Teekhasaenee and Ritch, 1997, Vidaurri-de la Cruz H, 2003), intracerebral vascular or other malformations leading to seizures and cognitive delay (Hall et al., 2007, Shields et al., 2011, Tsuruta et al., 1999, Vidaurri-de la Cruz H, 2003), overgrowth of soft tissues or limbs (Chhajed et al., 2010, Jeon et al., 2013, Vidaurri-de la Cruz H, 2003), and melanoma. The latter can arise in choroid or conjunctiva, with melanocytoma of the optic disk also described (Krema et al., 2013, Shields et al., 2011, Teekhasaenee and Ritch, 1997, Tran and Zografos, 2005). Without an understanding of the genetic pathogenesis of the disease, targeted therapies for the congenital and neoplastic aspects of the disease have been impossible.

When considering the genetic basis of extensive dermal melanocytosis and of PPV, we hypothesized that these conditions could be the result of a postzygotic mutation in a member of the G-protein nucleotide binding protein alpha subunit family. This hypothesis was generated by the rare concurrent description of PPV with Sturge-Weber syndrome (SWS) (Chhajed et al., 2010; Vidaurri-de la Cruz et al., 2003), a vascular disorder with no pigmentary phenotype recently found to be the result of postzygotic mosaicism for activating mutations in *GNAQ* (Shirley et al., 2013). We also hypothesized that an identical mutation could be present in both types of birthmarks in PPV, secondary to a single mutation in a pluripotent progenitor cell.

## Results

**Figure 2:**
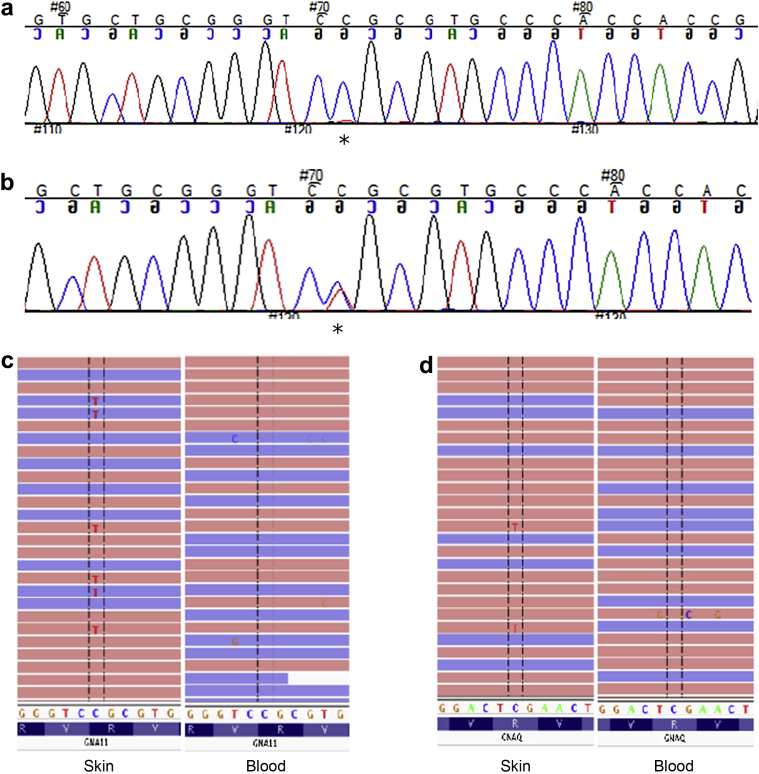
**Sequencing results demonstrating mosaic *GNA11* and *GNAQ* mutations.** (**a**) Sanger sequencing of skin biopsy showing a very low peak in *GNA11* at position c.547C>T (p.Arg183Cys) (asterisk). (**b**) Sanger sequencing of the same skin biopsy deoxyribonucleic acid after restriction enzyme digest of the normal allele, and hemi-nested amplification, revealing the mutation (asterisk). (**c**) Targeted next-generation sequencing showing low allele percentage mutations in skin but undetectable in blood, *GNA11* c.547C>T (p.Arg183Cys) in 5% of reads. (**d**) *GNAQ* c.548G>A (p.Arg183Gln) in 6% of reads from skin but undetectable in blood.

Missense *GNA11* or *GNAQ* mutations were found in 8 of 11 patients tested. In each case, these mutations were detected in affected skin (and in one case ocular tissue) at very low levels and were undetectable in blood, indicating postzygotic mosaicism (Table 1). When levels of mutant allele <1% were detected in blood, these could not be distinguished from background noise despite the substantial depth of coverage (Supplementary Table S2 online) and were therefore called as wild type (WT). In the three patients with extensive dermal melanocytosis, we identified mutations in *GNAQ* in two, one c.548G>A, p.R183Q, and one c.626A>C, Q209P. In the eight patients with PPV, we identified somatic *GNA11* mutations in four, three at position c.547C>T, p.R183C, and one novel mutation in the same codon c.547C>A, p.R183S. and *GNAQ* mutations in two, both at position c.548G>A, p.R183Q (Figure 2). Importantly there was conservation of mutation between pigmentary and vascular lesions in each patient for whom more than one skin biopsy was available (Table 1 and Supplementary Table S2). Measured percentage of mosaicism in skin samples was lowest at 1.5% using next-generation sequencing mutant allele count as a percentage of the total number of reads (Supplementary Table S2). None of these mutations in *GNA11* and *GNAQ* is described in the largest current population database (Exome Aggregation Consortium, 2015).

**Figure 3:**
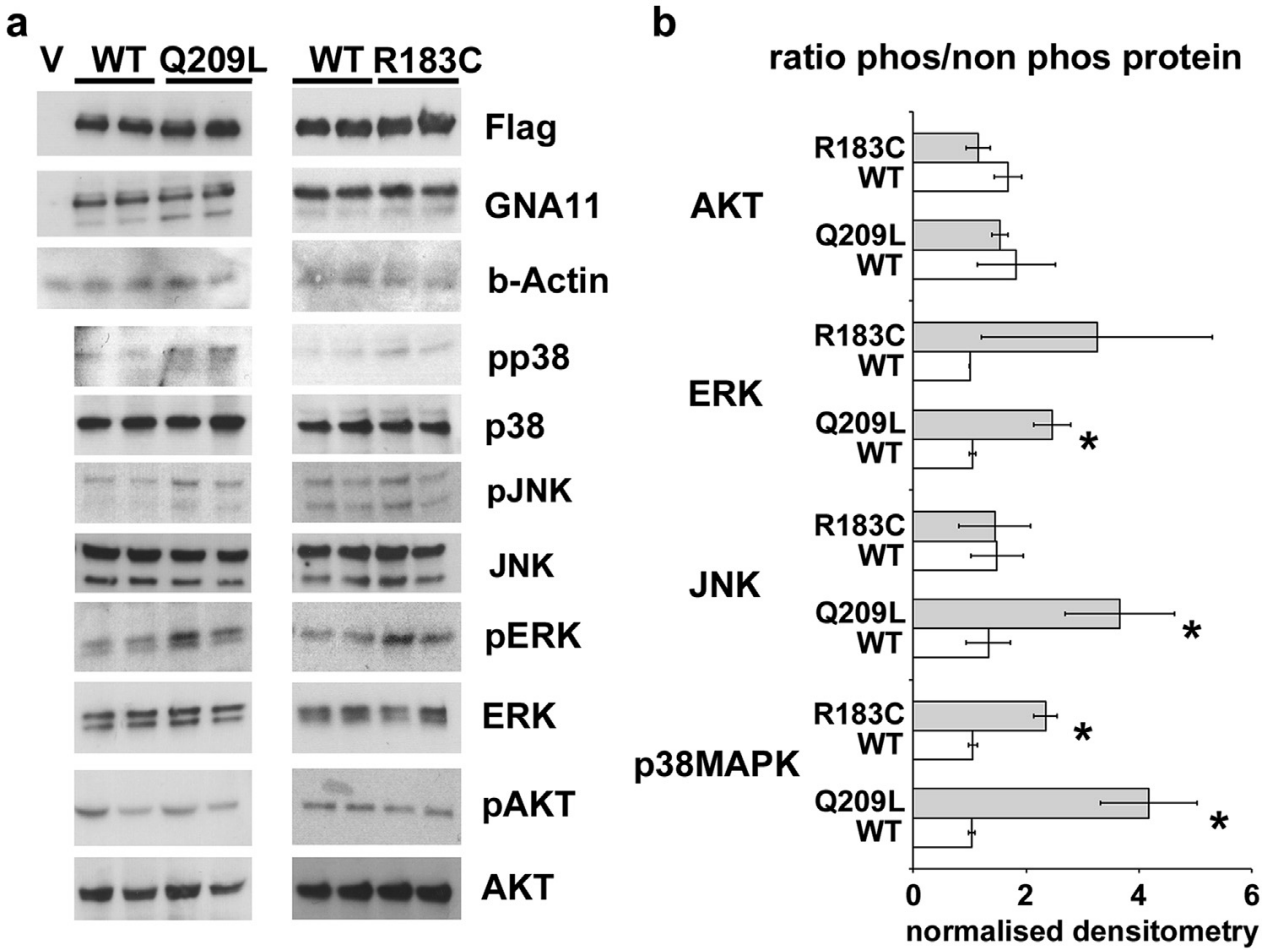
**Mutant *GNA11* leads to activation of downstream signaling pathways.** (**a**) Western blot for Flag, total GNA11, and the phosphorylated and total p38, JNK, ERK and AKT in total protein lysates from HEK293T cells transfected with either vector, wild-type *GNA11* (WT), or one of two mutants R183C or Q209L mutant *GNA11*. (**b**) Ratio of phosphorylated to total p38, JNK, ERK, and AKT normalized by actin loading control. Bars indicate 1 standard deviation. ∗*P* < 0.05, unpaired t-test.

For the in vitro transfection experiments, HEK293 cells were transfected with vector, WT *GNA11*, or mutant *GNA11* constructs encoding R183C and Q209L. Figure 3 shows results of western blots for Flag, total GNA11, and the phosphorylated and total p38, JNK, ERK and AKT in total protein lysates from these cells. Analysis of the ratio of phosphorylated to total p38, JNK, ERK and AKT normalized by the actin loading control (from two replicates, pooled results of WT compared to those of mutant) demonstrated increased phosphorylation and therefore activation of the p38 MAPK pathway by the R183C mutant and of ERK, p38 MAPK, and JNK pathways by Q209L, in keeping with previous results for Q209L (Li et al., 2014). These results were significant at the 0.05 level using a one-tailed *t* test.

**Figure 4:**
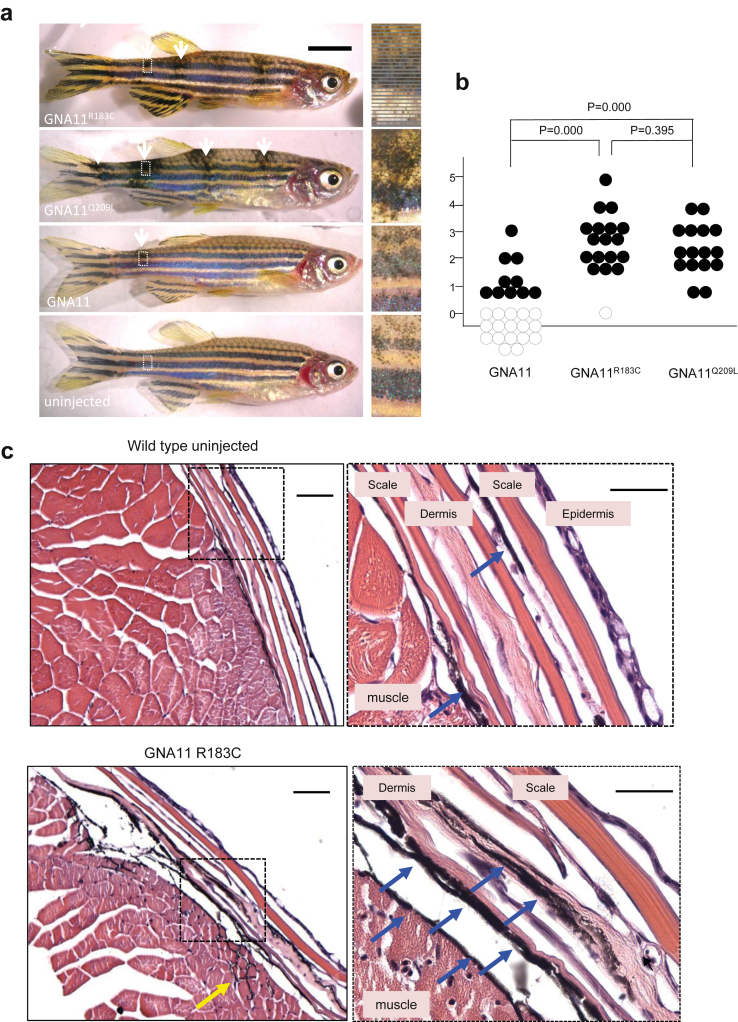
**Mosaic expression of *GNA11* promotes ectopic pigmentary lesions in zebrafish.** (**a**) Images of adult zebrafish mosaic for *GNA11, GNA11*^*R183C*^, *or GNA11*^*Q209L*^ expression. Large, ectopic pigmentary lesions are indicated next to white arrows. Dashed box indicates zoomed areas that show detail of pigmentary lesions. (**b**) Numbers of pigmentary lesions per fish expressing *GNA11, GNA11*^*R183C*^, or *GNA11*^*Q209L*^. Dark circles indicate ectopic pigmentary lesions. White circles indicate fish without pigmentary lesions. (**c**) Histology hematoxylin and eosin staining of a wild-type and *GNA11*^*R183C*^ zebrafish skin at ×100 and ×400 magnification. Melanocytes are clearly visible in the dermis by the black melanin (blue arrows), frequently also in the epidermis (not shown) and in a few cases within underlying muscle (yellow arrow).

*GNA11*^*R183C*^*-* and *GNA11*^*Q209L*^-expressing zebrafish exhibited dark cutaneous patches of melanocytes by 1 month. When the mutant transgenic zebrafish reached adulthood at 3 months, nearly all injected fish (n = 17/18 for R183C and n = 16/16 for Q209L) developed large and clearly visible pigmentary lesions (Figure 4a and b), recapitulating the human phenotype of dermal melanocytosis. Wild-type *GNA11* expression in melanocytes also produced mosaic animals with some pigmentary lesions (n = 10/27); however, these were usually smaller and less dark, suggesting that the melanocyte lineage is highly sensitive to GNA11-coupled receptor signaling. Histopathology revealed many extra melanocytes in both the epidermis (over the scales, not shown) and the dermis in the pigmentary lesion of mutant *GNA11*^*R183C*^ injected fish, with occasional involvement of the underlying muscle (Figure 4c).

## Discussion

We sought to differentiate a subgroup of the common birthmark dermal melanocytosis in infants who are at risk of extra-cutaneous complications, by elucidating the underlying genetic cause. We show here that extensive dermal melanocytosis and PPV are genetic conditions associated with postzygotic mutations in genes encoding Gα subunits of heterotrimeric G proteins. Specifically, postzygotic mutations in *GNAQ* have been found in extensive dermal melanocytosis, and mutations in *GNA11* or *GNAQ* have been found in PPV types I, II (cesioflammea), IV, and V (cesiomarmorata), as well as the unclassifiable achromico-melano-marmorata type. Type IV is considered to be exceptionally rare and was not tested because no samples were available. Type III (spilorosea) is distinguished clinically from all other types of PPV by the absence of dermal melanocytosis, and because only one patient from this group was tested (and was WT), we cannot yet conclude anything about the genetic etiology of this type. Of note, the percentage mosaicism was often very low, the result of very few mutant cells within a skin biopsy of a completely macular birthmark, thus emphasizing the need for adequately sensitive detection techniques in this type of genetic disease. A possible alternative explanation for our findings is that these patients are somehow genetically predisposed to somatic mutations in the same genes, and this is particularly relevant for those in whom we have only been able to obtain one sample. Although this cannot be entirely excluded, it is a much less convincing explanation of the results than postzygotic mosaicism, as we discovered different mutations in different patients, but the same mutation was present in more than one location when two samples were obtained from one individual. As candidate gene sequencing was performed in this study, we cannot exclude the presence of further mutation in other genes. Further exome sequencing could be performed to look for secondary mutations.

True embryonic mosaicism has not yet been described for *GNA11* mutations. Isolated somatic *GNA11* mutations have been found in uveal melanoma and single blue nevi (a lesion distinct from dermal melanocytosis both clinically and histologically) (Van Raamsdonk et al., 2010). In contrast to our findings in PPV, the prevalence of codon 183 mutations in single melanomas and blue nevi was much lower than of codon 209 mutations (Van Raamsdonk et al., 2010). Postzygotic mosaicism for *GNAQ* mutations has been described as the major cause of SWS, a purely vascular disorder with no pigmentary phenotype. Somatic mutations in *GNAQ* are frequent in uveal melanoma and single blue nevi (Van Raamsdonk et al., 2009). Therefore, our findings not only describe *GNA11* mosaicism but also extend the phenotypic spectrum of human *GNAQ* mosaicism from SWS (vascular only), through to PPV (vascular and pigmentary), to extensive dermal melanocytosis (pigmentary only). Exactly how the same mutation can generate these three distinct phenotypes is not yet clear.

Codon 183 in both *GNA11* and *GNAQ* is located within the guanosine triphosphate binding region of the human Gα subunit, a region required for hydrolysis of guanosine triphosphate to guanosine diphosphate and an essential step for inactivation of G-protein coupled receptor signaling. Therefore, these mutations lead to decreased function of guanosine triphosphatase and to constitutive activation of downstream effector pathways. We have shown that the most common mutation at codon 183 of *GNA11* activates the downstream p38 MAPK pathway in human cells, whereas the codon 209 mutant (not found in this cohort in *GNA11* but in one patient in *GNAQ*) activates p38 MAPK, JNK and ERK pathways, broadly supporting previous findings of effects of mutations in both *GNA11* (Li et al., 2014, Van Raamsdonk et al., 2010) and *GNAQ* (Van Raamsdonk et al., 2010).

Of interest, different mutations in *GNA11* in the germline leading to altered sensitivity of cells to extracellular calcium concentrations have been described in autosomal dominant hypoparathyroidism and hypocalciuric hypercalcemia, respectively (Nesbit et al., 2013). One of these mutations (*GNA11*^*R60L*^) has been characterized as less activating than *GNA11*^*Q209L*^ (Li et al., 2014). This pattern of less severe mutations being supportable in the germline, whereas more severe mutations can survive only by mosaicism, once again confirms Happle’s established theory (Happle, 1987). Patients with PPV and SWS have not been documented to have abnormalities of calcium homeostasis; however, intravascular calcification can be a feature of neurological abnormalities in PPV, which could hypothetically involve localized mosaic calcium imbalances. This is an area that merits future careful research.

A causal link between our genetic findings and mechanism is strongly supported by the mosaic zebrafish models demonstrated here, in which expression of *GNA11*^*R183C*^ or *GNA11*^*Q209L*^ leads to ectopic and increased numbers of melanocytes in the epidermal and dermal layers, recapitulating the human phenotype of dermal melanocytosis. Overexpression of WT *GNA11* in melanocytes also leads to a low level of pigmentary mosaicism in zebrafish, underscoring the importance of close regulation of G-protein coupled receptor signaling in the melanocyte lineage. No other mosaic animal models of *GNA11* gain-of-function mutations exist; however, further conformation of our findings comes from germline hypermorphic alleles of *GNA11*, which lead to increased cutaneous pigmentation in the murine *Dsk* phenotype (Van Raamsdonk et al., 2004).

We found no evidence for the now-retracted theory of twin spotting or didymosis. Rather, we have shown that a single somatic mutation in *GNA11* or *GNAQ* is responsible for both types of cutaneous lesion in PPV, mirroring closely the pathogenesis of the disparate birthmarks seen in *HRAS* mosaicism (Groesser et al., 2013). We propose that extensive dermal melanocytosis and PPV are members of an expanding group of heterotrimeric G-protein alpha subunit gene mosaic conditions, joining McCune-Albright syndrome (Weinstein et al., 1991), caused by mosaicism for gain-of-function mutations in *GNAS*, and SWS, caused by mosaic gain-of-function mutations in *GNAQ* (Shirley et al., 2013). We also propose that *GNAQ* mosaicism forms a spectrum from SWS, through PPV, to extensive dermal melanocytosis. This knowledge will allow accurate clinical molecular diagnosis of a subset of extensive dermal melanocytosis and of PPV, leading to identification of neonates at risk for serious complications associated with these birthmarks. Importantly, it will also pave the way for therapeutic options in these children.

## Materials and Methods

All human and animal studies were approved by the authors' institutions' research ethics review boards. Declaration of Helsinki protocols were followed, and all patients gave written informed consent. Eleven patients from four international centers were recruited with written consent for genetic research, approved by the local research ethics committees. Patients were classified by clinical phenotype: three with extensive or atypical dermal melanocytosis (which was defined as involving areas other than only the lumbosacral lesion) and eight with PPV, with representatives of subtypes I, II (cesioflammea), III (spilorosea), and V (cesiomarmorata) (Supplementary Table S1 and Figure 1), and also the unclassifiable achromico-melano-marmorata type (Boente Mdel et al., 2008). Blood samples and skin biopsies were taken from each patient, and in those patients with PPV, skin was biopsied from both the vascular and pigmentary birthmarks when possible.

### Selective amplification of mutant alleles of candidate genes *GNA11* and *GNAQ* for Sanger sequencing

Deoxyribonucleic acid (DNA) was extracted from whole blood and directly from fresh skin lesion samples using the DNeasy Blood and Tissue Kit (Qiagen, Düsseldorf, Germany) and from paraffin embedded tissue using the RecoverAll total nucleic acid extraction kit for formalin-fixed paraffin-embedded tissue (Life Technologies, Carlsbad, CA). To maximize detection of mutant alleles at low percentage mosaicism, we designed restriction enzyme digests of the normal allele at hotspots codons 183 and 209 of *GNA11* and 183 of *GNAQ* using validated methods (Kinsler et al., 2013) and Sanger sequencing. Primer sequences and restriction enzymes for each hotspot are given in Supplementary Table S3 (online). Touchdown polymerase chain reaction programs were used throughout, with 35 cycles for the first polymerase chain reaction and 25 for the second.

### Targeted deep sequencing of *GNA11* and *GNAQ* and data analysis

We performed targeted deep sequencing of the regions spanning mutations previously identified by selective amplification of mutant alleles in all samples for which DNA quality was sufficient (as determined by the presence of a band above 10 kb size on agarose gel). In subjects with no identified mutation, all coding regions of *GNAQ* and *GNA11* were screened using the same method. Targeted regions were amplified using custom intronic primers and standard long-range polymerase chain reaction protocols. Polymerase chain reaction products were purified and libraries were prepared using the Nextera XT DNA Sample Preparation kit (Illumina, Cambridge, UK). Samples were then pooled and sequenced on a MiSeq instrument (Illumina) according to the manufacturer’s recommendations for paired-end 150-bp reads. In-depth sequencing was performed to achieve a sequencing depth of at least 1,000 reads for all targeted coding bases and splice junctions. Identification of candidate variants was performed as described previously (Riviere et al., 2012). Briefly, all targeted bases were systematically screened to count all sites with at least one read not matching the reference sequence, using a base-quality threshold of 30. Candidate postzygotic variants were confirmed by at least one independent experiment in all DNA samples available from the patient.

### Zebrafish model

To test the causality and model the effects of the mutation, we injected zebrafish embryos with WT human *GNA11*, *GNA11*^*R183C*^, or *GNA11*^*Q209L*^ expressed from the melanocyte *mitfa* promoter and grew the genetically mosaic animals to adulthood. All zebrafish work was performed in accordance with United Kingdom Home Office Animals (Scientific Procedures) Act (1986) and approved by the University of Edinburgh Ethical Review Committee.

The human *GNA11* cDNA clone was purchased from Origene (purchased from NM_002067.1 precloned into an untagged pCMV6-XL4 vector, catalog number SC303115). Mutant GNA11Q209L (626a> t) was generated by site-directed genesis polymerase chain reaction with primers (forward: 5′-GATGTGGGGGGCCTGCGGTCGGAGCGGAGG-3′, reverse: 5′-CCTCCGCTCCGACCGCAGGCCCCCCACATC-3′). Mutant GNA11R183C (547c>t) was also generated with primers (forward: 5′-CGTGCTGCGGGTCTGCGTGCCCACCACCG-3′, reverse: 5′-CGGTGGTGGGCACGCAGACCCGCAGCACG-3′). The WT and mutant GNA11 cDNAs were cloned together with the zebrafish *mitfa* gene promoter into the pDestTol2CG2 expression vector using the Tol2kit gateway cloning method (Kwan et al., 2007), resulting in *mitfa*-GNA11, *mitfa*-GNA11Q^209L^ and *mitfa*-GNA11R^183C^ constructs. For selection purposes, these constructs contain an additional green fluorescent protein gene expressed from the heart *cml* promoter. Two nanoliters of mixed mitfa-GNA11 plasmid DNA and Tol2 mRNA (62.5 ng/μl and 70 ng/μl, respectively) was injected into one-cell stage zebrafish embryos. Zebrafish embryos with green fluorescent protein transgenic marker in the heart were selected and grown to adulthood.

Adult zebrafish were anesthetized in 50 mg/L tricaine solution and ×1 images from both sides of the fish were taken under a stereomicroscope. Dark patches of melanocyte lesions with areas larger than that of one scale were counted. Adult zebrafish were sacrificed by immersion in tricaine solution as directed by Home Office Schedule 1 methods. Zebrafish were then dissected in half transversely to increase penetration of the fixative and fixed in 4% freshly prepared paraformaldehyde (Electron Microscopy Sciences, Hatfield, PA) at 4 °C for 3 days. Samples were washed in phosphate buffered saline and decalcified in 0.5 M EDTA at pH 8 for 5 days before storage in 70% ethanol. Samples were embedded in paraffin wax and sectioned at 5-μm thickness. For hematoxylin and eosin staining, slides were deparaffinized in xylene twice for 5 minutes and then rehydrated through graded alcohol solutions (100%, 90%, 70%, 50% and 30% for 3 minutes each) and stopped in water. Slides were stained with Mayer hematoxylin and eosin after the standard hematoxylin and eosin staining procedure.

### Analysis of downstream effectors of GNA11 in human cell line in culture

To characterize the functional effect of mutations on downstream signaling pathways, HEK293T cells were transfected with vector, WT *GNA11*, or mutant *GNA11* constructs encoding R183C and Q209L. The Q209L was used as a positive comparator because it is a more studied mutation in *GNA11*. Flag-tagged GNA11 WT, R183C, and Q209L constructs and vector alone control (pDEST26, Invitrogen, Carlsbad, CA) were transfected into HEK293 cells using lipofectamine 2000 (Invitrogen), according to the manufacturer’s instructions. Cells were cultured for another 24 hours, and protein lysates were prepared by boiling in a denaturing SDS buffer (2% 2-mercaptoethanol, 2% SDS, 10mM Tris pH 7.5) for 10 minutes. Levels of GNA11 effector activation were assessed by western blot using the following antibodies at the following concentrations: Rabbit anti–p-Serine473 Akt (1/500; Cell Signaling Technology, Danvers, MA), rabbit anti-total Akt (1/1,000, Cell Signaling Technology), rabbit anti-phospho T180/Y182 p38 MAPK (1/500; Cell Signaling Technology), rabbit anti-Total p38 MAPK (1/500, Cell Signaling Technology), mouse anti-phospho T183/Y185 SAPK/JNK (1/500; Cell Signaling Technology), rabbit anti-total SAPK/JNK (1/500; Cell Signaling Technology), pT202/Y204 ERK (1/500; Cell Signaling Technology), and rabbit anti-total ERK 1,2 (1/1,000; Cell Signaling Technology), mouse anti-Flag (DYKDDDDK tag) (1/500; Cell Signaling Technology), rabbit anti-GNA11 (1/500; GeneTex, Irvine, CA), and mouse anti-GAPDH (1/2,000; Millipore, Watford, UK).

Primary antibody incubations were in TBST (100 mM Tris HCl, 0.2 M NaCl, 0.1% Tween-20 v/v) containing 5% bovine serum albumin (Sigma, Gillingham, United Kingdom) or 5% skimmed milk powder either overnight at 4 ^o^C or for 1–2 hours at room temperature, whereas secondary antibody incubations were performed in 5% skimmed milk powder for 1 hour at room temperature. The concentrations used were swine anti-rabbit HRP (DakoCytomation, Carpinteria, CA) 1:3,000 and rabbit anti-mouse HRP (DakoCytomation) 1:2,000. Protein was visualized using Luminol reagent (Santa Cruz Biotechnologies, Dallas, TX). Densitometry of bands was performed using thresholded images in the ImageJ suite (imagej.nih.gov/ij/).

## ORCIDs

Veronica Kinsler: http://orcid.org/0000-0001-6256-327X

Pierre Vabres: http://orcid.org/0000-0001-8693-3183

## Conflict of Interest

The authors state no conflicts of interest.

## Figures and Tables

**Table 1:** Clinical phenotype and *GNA11 and GNAQ* genotypes with percentage mosaicism (where available) of different samples from eight patients with PPV (patients 1–8) and three patients with extensive dermal melanocytosis with no vascular phenotype (patients 9–11), showing postzygotic mosaicism

Patient	Diagnosis	Pigmentary skin lesion	Vascular skin lesion	Blood	Other samples	Result
1	PPV unclassifiable (I) type: capillary malformation on face (including forehead), trunk; pigmentary lesions on limbs, trunk; linear sebaceous nevus on scalp; linear woolly hair; glaucoma; no overgrowth or neurological abnormalities		*GNA11* c.547C>T, p.R183C 5.3%	WT 0.0%		*GNA11* p.R183C mosaic
2	PPV cesioflammea (II) type: capillary malformation on face (including forehead); scleral melanocytosis (no dermal melanocytosis); no overgrowth or ophthalmological or neurological abnormalities		*GNA11* c.547C>A, p. R183S (novel)		Buccal swab *GNA11* c.547C>A, p.R183S 7.9% Normal skin WT	*GNA11* p.R183S mosaic
3	PPV cesioflammea (II) type: capillary malformation on face (including forehead), trunk, limbs; nevus anemicus; dermal melanocytosis on trunk; scleral melanocytosis; bilateral glaucoma; renal vascular hypertension; hemihypertrophy; macrocephaly; CNS MRI reveals enlarged supratentorial subarachnoid space, enlarged lateral ventricles, increased arachnoid vascular network, asymmetrical venous flow, absence of pial angioma		*GNAQ* c.548G>A, p.R183Q 6.4%	WT 0.0%	Pigmented ocular tissue *GNAQ* c.548G>A, p.R183Q 11.0%	*GNAQ* p.R183Q mosaic
4	PPV cesioflammea (II) type: capillary malformation on face (including forehead), trunk, limbs; dermal melanocytosis on trunk, limbs; growth delay; small teeth; developmental delay; no overgrowth or ophthalmological or radiological neurological abnormalities		*GNAQ* c.548G>A, p.R183Q 5.0%	WT 0.0%		*GNAQ* p.R183Q mosaic
5	PPV cesioflammea (II) type: capillary malformation on face (including forehead), trunk, foot; dermal melanocytosis on face (including forehead), trunk, limbs; seizures; moderate global developmental delay; overgrowth; bilateral glaucoma; Sturge-Weber syndrome-like pial angioma on CNS MRI	WT 0.0%	WT 0.0%	WT 0.0%		WT
6	PPV achromico-melano-marmorata type (unclassifiable in both classifications): hyper- and hypo-pigmentary and reticulate vascular lesions affecting trunk, limbs; undergrowth of one leg (both legs affected by vascular lesions); no ophthalmological or neurological abnormalities		*GNA11* c.547C>T, p.R183C	WT		*GNA11* p.R183C mosaic
7	PPV spilorosea type (III): pigmentary and vascular lesions affecting trunk, limbs but not face; no overgrowth or ophthalmological or neurological abnormalities	WT 0.0%	WT 0.0%	WT 0.0%		WT
8	PPV cesiomarmorata type (V): pigmentary lesions on trunk; vascular lesions on face (including forehead), trunk, limbs; mild developmental delay, but CNS MRI shows periventricular leukomalacia consistent with premature delivery; no overgrowth or ophthalmological abnormalities	*GNA11* c.547C>T, p.R183C 15.5%	*GNA11* c.547C>T, p.R183C 9.6%	WT 0.3% same mutation		*GNA11* p.R183C mosaic
9	Extensive and multiple dermal melanocytosis affecting trunk and limbs; no overgrowth or ophthalmological or neurological abnormalities	*GNAQ* c.548G>A, p.R183Q 2%	N/A	WT 0.1% same mutation		*GNAQ* p.R183Q mosaic
10	One large (>20 cm) dark, well-defined, persistent flank dermal melanocytosis; no overgrowth or ophthalmological or neurological abnormalities	*GNAQ* c.626A>C, p.Q209P 5.7%	N/A	WT 0.1% same mutation		*GNAQ* p.Q209P mosaic
11	Extensive and multiple dermal melanocytosis affecting trunk; no overgrowth or ophthalmological or neurological abnormalities	WT 0.0%	N/A	WT 0.0%		WT

Abbreviations: CNS, central nervous system; MRI, magnetic resonance imaging; N/A, not applicable; PPV, phakomatosis pigmentovascularis; WT, wild type.

For coordinates and exact wild-type and mutant allele numbers, see Supplementary Table S2 online. When mutant allele detection is <1%, we cannot confidently distinguish this from background noise despite the depth of coverage; therefore, these samples are assigned as WT.

## Supplementary Material

Supplementary Tables S1–S3 and Supplementary References

## References

[bib1] Boente Mdel C., Obeid R., Asial R.A., Bibas-Bonet H., Coronel A.M., Happle R. (2008). Cutis tricolor coexistent with cutis marmorata telangiectatica congenita: “phacomatosis achromico-melano-marmorata”. Eur J Dermatol.

[bib2] Chhajed M., Pandit S., Dhawan N., Jain A. (2010). Klippel-Trenaunay and Sturge-Weber overlap syndrome with phakomatosis pigmentovascularis. J Pediatr Neurosci.

[bib3] Cordova A. (1981). The Mongolian spot: a study of ethnic differences and a literature review. Clin Pediatr (Phila).

[bib4] Danarti R., Happle R. (2003). Paradominant inheritance of twin spotting: phacomatosis pigmentovascularis as a further possible example. Eur J Dermatol.

[bib5] Exome Aggregation Consortium. http://exac.broadinstitute.org. 2015 (accessed June 2015).

[bib6] Groesser L., Herschberger E., Sagrera A., Shwayder T., Flux K., Ehmann L. (2013). Phacomatosis pigmentokeratotica is caused by a postzygotic HRAS mutation in a multipotent progenitor cell. J Invest Dermatol.

[bib7] Gupta D., Thappa D.M. (2013). Mongolian spots—a prospective study. Pediatr Dermatol.

[bib8] Hall B.D., Cadle R.G., Morrill-Cornelius S.M., Bay C.A. (2007). Phakomatosis pigmentovascularis: Implications for severity with special reference to Mongolian spots associated with Sturge-Weber and Klippel-Trenaunay syndromes. Am J Med Genet A.

[bib9] Hanson M., Lupski J.R., Hicks J., Metry D. (2003). Association of dermal melanocytosis with lysosomal storage disease: clinical features and hypotheses regarding pathogenesis. Arch Dermatol.

[bib36] Happle R. (1987). Lethal genes surviving by mosaicism: a possible explanation for sporadic birth defects involving the skin. J Am Acad Dermatol.

[bib10] Happle R. Mosaicism in human skin. Berlin Heidelberg: Springer-Verlag; 2013.

[bib12] Hasegawa Y., Yasuhara M. (1979). A variant of phakomatosis pigmentovascularis. Skin Res.

[bib13] Henry C.R., Hodapp E., Hess D.J., Blieden L.S., Berrocal A.M. (2013). Fluorescein angiography findings in phacomatosis pigmentovascularis. Ophthalmic Surg Lasers Imaging Retina.

[bib14] Igawa H.H., Ohura T., Sugihara T., Ishikawa T., Kumakiri M. (1994). Cleft lip mongolian spot: mongolian spot associated with cleft lip. J Am Acad Dermatol.

[bib15] Jeon S.Y., Ha S.M., Ko D.Y., Hong J.W., Song K.H., Kim K.H. (2013). Phakomatosis pigmentovascularis Ib with left-sided hemihypertrophy, interdigital gaps and scoliosis: a unique case of phakomatosis pigmentovascularis. J Dermatol.

[bib16] Kinsler V.A., Thomas A.C., Ishida M., Bulstrode N.W., Loughlin S., Hing S. (2013). Multiple congenital melanocytic nevi and neurocutaneous melanosis are caused by postzygotic mutations in codon 61 of NRAS. J Invest Dermatol.

[bib17] Krema H., Simpson R., McGowan H. (2013). Choroidal melanoma in phacomatosis pigmentovascularis cesioflammea. Can J Ophthalmol.

[bib18] Kwan K.M., Fujimoto E., Grabher C., Mangum B.D., Hardy M.E., Campbell D.S. (2007). The Tol2kit: a multisite gateway-based construction kit for Tol2 transposon transgenesis constructs. Dev Dyn.

[bib19] Li D., Opas E.E., Tuluc F., Metzger D.L., Hou C., Hakonarson H. (2014). Autosomal dominant hypoparathyroidism caused by germline mutation in GNA11: phenotypic and molecular characterization. J Clin Endocrinol Metab.

[bib20] Nesbit M.A., Hannan F.M., Howles S.A., Babinsky V.N., Head R.A., Cranston T. (2013). Mutations affecting G-protein subunit alpha11 in hypercalcemia and hypocalcemia. N Engl J Med.

[bib21] Ota M., Kawamura T., Ito N. (1947). Phakomatosis pigmentovascularis. Jpn J Dermatol.

[bib22] Riviere J.B., Mirzaa G.M., O'Roak B.J., Beddaoui M., Alcantara D., Conway R.L. (2012). De novo germline and postzygotic mutations in AKT3, PIK3R2 and PIK3CA cause a spectrum of related megalencephaly syndromes. Nat Genet.

[bib23] Shields C.L., Kaliki S., Livesey M., Walker B., Garoon R., Bucci M. (2013). Association of ocular and oculodermal melanocytosis with the rate of uveal melanoma metastasis: analysis of 7872 consecutive eyes. JAMA Ophthalmol.

[bib24] Shields C.L., Kligman B.E., Suriano M., Viloria V., Iturralde J.C., Shields M.V. (2011). Phacomatosis pigmentovascularis of cesioflammea type in 7 patients: combination of ocular pigmentation (melanocytosis or melanosis) and nevus flammeus with risk for melanoma. Arch Ophthalmol.

[bib25] Shirley M.D., Tang H., Gallione C.J., Baugher J.D., Frelin L.P., Cohen B. (2013). Sturge-Weber syndrome and port-wine stains caused by somatic mutation in GNAQ. N Engl J Med.

[bib26] Teekhasaenee C., Ritch R., Rutnin U., Leelawongs N. (1990). Ocular findings in oculodermal melanocytosis. Arch Ophthalmol.

[bib27] Teekhasaenee C., Ritch R. (1997). Glaucoma in phakomatosis pigmentovascularis. Ophthalmology.

[bib28] Tran H.V., Zografos L. (2005). Primary choroidal melanoma in phakomatosis pigmentovascularis IIa. Ophthalmology.

[bib29] Tsuruta D., Fukai K., Seto M., Fujitani K., Shindo K., Hamada T. (1999). Phakomatosis pigmentovascularis type IIIb associated with moyamoya disease. Pediatr Dermatol.

[bib30] Van Raamsdonk C.D., Bezrookove V., Green G., Bauer J., Gaugler L., O'Brien J.M. (2009). Frequent somatic mutations of GNAQ in uveal melanoma and blue naevi. Nature.

[bib31] Van Raamsdonk C.D., Fitch K.R., Fuchs H., de Angelis M.H., Barsh G.S. (2004). Effects of G-protein mutations on skin color. Nat Genet.

[bib32] Van Raamsdonk C.D., Griewank K.G., Crosby M.B., Garrido M.C., Vemula S., Wiesner T. (2010). Mutations in GNA11 in uveal melanoma. N Engl J Med.

[bib33] Vidaurri-de la Cruz H., Tamayo-Sanchez L., Duran-Mckinster C., Orozco-Covarrubias M.L., Ruiz-Maldonado R. (2003). Phakomatosis pigmentovascularis II A and II B: clinical findings in 24 patients. J Dermatol.

[bib35] Weinstein L.S., Shenker A., Gejman P.V., Merino M.J., Friedman E., Spiegel A.M. (1991). Activating mutations of the stimulatory G protein in the McCune-Albright syndrome. N Engl J Med.

