# Supplementary material for: Mosaic Activating Mutations in GNA11 and GNAQ Are Associated with Phakomatosis Pigmentovascularis and Extensive Dermal Melanocytosis
Source: J Invest Dermatol. 2016 Apr;136(4):770–8. doi: 10.1016/j.jid.2015.11.027 (PMC4803466; doi:10.1016/j.jid.2015.11.027)
Supplement: Supplementary Tables S1–S3 and Supplementary References [file mmc1.pdf]

Supplementary table 1

Published clinical classifications of phakomatosis pigmentovascularis, based on the types of vascular and pigmentary birthmarks

Vascular lesions	Pigmentary lesions	Classification 1(1)	Classification 2(2)
Capillary malformation (port wine stain/naevus flammeus)	Linear epidermal naevus	Type I	Not included in this classification
Capillary malformation (port wine stain/naevus flammeus)	Dermal melanocytosis (Mongolian blue spot)	Type II	‘Cesioflammea’ type
Capillary malformation (port wine stain/naevus flammeus in classification 1, pale pink telengiectatic naevus in classification 2)	Naevus spilus	Type III	‘Spilorosea’ type
Capillary malformation (port wine stain/naevus flammeus)	Dermal melanocytosis (Mongolian blue spot) and naevus spilus	Type IV	Extremely rare, unclassifiable in this classification
Capillary malformation (cutis marmorata telengiectatica congenita)	Dermal melanocytosis (Mongolian blue spot)	Type V	‘Cesiomarmorata’ type

Supplementary table 2

Mutant allele count and percentage of mosaicism for each of the samples tested by the next generation sequencing method. Please note where percentage mosaicism is lower than 1% it is not possible to distinguish this from background noise, and therefore these are considered as wild-type.

Patient # in table 1	gDNA (hg19)	cDNA	aa change	Reference allele count	Alternative allele count	Total allele count	Percent mosaicism	Sample origin	Comments	Sequenced first by Sanger/ digest method
1	chr19:g.3115012C>T	NM_002067.2:c.547C>T	*GNA11* p.Arg183Cys	3749	209	3958	5.3	Skin		No
1	chr19:g.3115012C>T	NM_002067.2:c.547C>T	*GNA11* p.Arg183Cys	1349	0	1349	0.0	Blood		No
2	chr19:g.3115012C>A	NM_002067.2:c.547C>A	*GNA11* p.Arg183Ser	6018	515	6533	7.9	Cheek swab		Yes
3	chr9:g.80412493C>T	NM_002072.3:c.548G>A	GNAQ p.Arg183Gln	29396	2005	31401	6.4	Skin		Yes
3	chr9:g.80412493C>T	NM_002072.3:c.548G>A	GNAQ p.Arg183Gln	3870	0	3870	0.0	Blood		Yes
3	chr9:g.80412493C>T	NM_002072.3:c.548G>A	GNAQ p.Arg183Gln	24044	2983	27027	11.0	Ocular tissue		Yes
4	chr9:g.80412493C>T	NM_002072.3:c.548G>A	GNAQ p.Arg183Gln	16439	858	17297	5.0	Skin		No
4	chr9:g.80412493C>T	NM_002072.3:c.548G>A	GNAQ p.Arg183Gln	33632	8	33640	0.0	Blood		No
5	-	-	-	-	-	-	-	Skin	No mutation found in *GNA11* or *GNAQ*	No
6	chr19:g.3115012C>T	NM_002067.2:c.547C>T	*GNA11* p.Arg183Cys					Skin	MiSeq failed	Yes
7	-	-	-	-	-	-	-	Skin	No mutation found in *GNA11* or *GNAQ*	Yes
8	chr19:g.3115012C>T	NM_002067.2:c.547C>T	*GNA11*	16631	3054	19685	15.5	Skin		Yes

			p.Arg183Cys							
8	chr19:g.3115012C>T	NM_002067.2:c.547C>T	*GNA11* p.Arg183Cys	7861	834	8695	9.6	Skin		Yes
8	chr19:g.3115012C>T	NM_002067.2:c.547C>T	*GNA11* p.Arg183Cys	8163	25	8188	0.3	Blood		Yes
8	chr19:g.3115012C>T	NM_002067.2:c.547C>T	*GNA11* p.Arg183Cys	10727	35	10762	0.3	Blood		Yes
9	chr9:g.80412493C>T	NM_002072.3:c.548G>A	GNAQ p.Arg183Gln	16670	332	17002	2.0	Skin		Yes
9	chr9:g.80412493C>T	NM_002072.3:c.548G>A	GNAQ p.Arg183Gln	6212	9	6221	0.1	Blood		
10	chr9:g.80409488T>G	NM_002072.2:c.626A>C	GNAQ p.Gln209Pro	21187	1269	22456	5.7	Skin		No
10	chr9:g.80409488T>G	NM_002072.2:c.626A>C	GNAQ p.Gln209Pro	42942	6	42948	0.0	Blood		
11	-	-	-	-	-	-	-	Skin	No mutation found in *GNA11* or *GNAQ*	No

Supplementary table 3

Primer sequences and restriction enzymes for selective amplification of mutant alleles at hotspots in *GNA11* and *GNAQ*

**Hotspot**	**First and second PCR forward primer**	**First PCR reverse primer**	**Second PCR reverse primer**	**Restriction enzyme**
GNA11 codon 183	TACCTGACCGACGTTGACC	GGTGGTCTCAAACTCCTGGA	ACACCGGGCAAATGAGC	BstUI (New England BioLabs®)
GNA11 codon 209	TGGTGGATGTGGGGGGCCAGTGGTC	CCGGCGCACCATCGAA	CTTGGCAGGTGGGGAAGG	BsrI (New England BioLabs®)
GNAQ codon 183	TCCCTTTCCGTAGACAGCTT	TGAAGCCTACACATGATTCCAG	TGAAGCCTACACATGATTCCAG	HinfI (New England BioLabs®)
GNAQ codon 209	TCATGATGTGTTACCCAGAATGT	CCATTCCCCACACCCTACTT		Not used due to difficulties in designing site

Supplementary references

1. Hasegawa Y, Yasuhara, M. A variant of phakomatosis pigmentovascularis. *Skin Research.* 1979;21(178-86.
2. Happle R. Phacomatosis pigmentovascularis revisited and reclassified. *Archives of Dermatology.* 2005;141(3):385-8.
